# Early alteration of peripheral blood lymphocyte subsets as a risk factor for delirium in critically ill patients after cardiac surgery: A prospective observational study

**DOI:** 10.3389/fnagi.2022.950188

**Published:** 2022-09-01

**Authors:** Xiao Li, Wei Cheng, Jiahui Zhang, Dongkai Li, Fei Wang, Na Cui

**Affiliations:** ^1^Department of Critical Care Medicine, Peking Union Medical College Hospital, Chinese Academy of Medical Sciences (CAMS), Beijing, China; ^2^Department of Laboratory, Peking Union Medical College Hospital, Chinese Academy of Medical Sciences (CAMS), Beijing, China; ^3^State Key Laboratory of Complex Severe and Rare Diseases, Peking Union Medical College Hospital, Beijing, China

**Keywords:** delirium, cardiac surgery, critical care, risk factors, CD4+ T cells

## Abstract

**Objective:**

There is a high incidence of delirium among patients with organ dysfunction undergoing cardiac surgery who need critical care. This study aimed to explore the risk factors for delirium in critically ill patients undergoing cardiac surgery and the predictive value of related risk factors.

**Methods:**

We conducted a prospective observational study on adult critically ill patients who underwent cardiac surgery between January 2019 and August 2021. Patients were consecutively assigned to delirium and non-delirium groups. Univariate analysis and multivariate logistic analysis were used to determine the risk factors for delirium. Receiver operating characteristic curves and a nomogram were used to identify the predictive value of related risk factors.

**Results:**

Delirium developed in 242 of 379 (63.9%) participants. Acute Physiology and Chronic Health Evaluation II (APACHE II) and Sequential Organ Failure Assessment (SOFA) scores were 14.2 ± 5.6 and 18 ± 8.4, respectively. Patients with delirium had longer cardiopulmonary bypass time (149.6 ± 59.1 vs. 126.7 ± 48.5 min, *p* < 0.001) and aortic cross-clamp time (98.7 ± 51.5 vs. 86.1 ± 41.6 min, *p* = 0.010) compared with the non-delirium group. The area under the curve was 0.824 for CD4^+^ T cell count and 0.862 for CD4/CD8 ratio. Multivariate analysis demonstrated that age [odds ratio (OR) 1.030, *p* = 0.038], duration of physical restraint (OR 1.030, *p* < 0.001), interleukin-6 (OR 1.001, *p* = 0.025), CD19^+^ B cell count (OR 0.996, *p* = 0.016), CD4^+^ T cell count (OR 1.005, *p* < 0.001) and CD4/CD8 ratio (OR 5.314, *p* < 0.001) were independent risk factors for delirium. A nomogram revealed that age, cardiopulmonary bypass duration, CD4^+^ T cell count and CD4/CD8 ratio were independent predictors of delirium.

**Conclusion:**

Age, duration of physical restraint, CD4^+^ T cell count and CD4/CD8 ratio were reliable factors for predicting delirium in critically ill patients after cardiac surgery. The receiver operating characteristic curves and nomogram suggested a potential role for CD4^+^ T cells in mediating potential neuroinflammation of delirium.

## Introduction

Surgery can be a life-saving procedure but significant complications may occur after routine procedures, especially in older and frailer patients after common procedures such as cardiac surgery. The burden of comorbidities among patients undergoing cardiac surgery is increasing. This brings a high risk of postoperative complications that increase treatment costs and reduce life expectancy and quality of life. One study showed a 57% rate of postoperative complications among patients who underwent cardiac surgery (Group ISOS, 2016). Up to a third of patients experience major postoperative morbidity with organ dysfunction that requires life-sustaining therapy in the intensive care unit (ICU) (Hein et al., 2006). Neurological complications after cardiac surgery are of particular concern, and may be devastating in terms of quality of life and societal economic burden (Martin et al., 2012; Vasilevskis et al., 2018).

Delirium or acute neurocognitive disorder is a complex neuropsychiatric syndrome characterized by acute and fluctuating changes in cognition and consciousness (Hayhurst et al., 2016). It has been noted in up to 47% of patients undergoing cardiac surgery and up to 80% of ICU patients (Morandi et al., 2014; Vallabhajosyula et al., 2017). Delirium often predisposes to poor cognitive recovery, functional decline, nursing home admission, and even death (Witlox et al., 2010; Hayhurst et al., 2016; Cortés-Beringola et al., 2021). Delirium is an indicator of adverse patient consequences, and its duration is associated with mortality, although it remains unclear if the relationship is causal.

The role of inflammation and its effector cells (circulating leukocytes) in the development of delirium in patients undergoing cardiac surgery remains poorly understood. Cardiopulmonary bypass (CPB), which is used extensively in cardiac surgery, has often been suggested to stimulate inflammation that is responsible for significant but poorly defined changes in leukocytes (Gawdat et al., 2017). The present study investigated the role of imbalanced peripheral immunity, demonstrated by peripheral lymphocyte counts and inflammatory markers, in the development of delirium in critically ill patients undergoing cardiac surgery. To help clinicians better understand the burden of delirium and predictive corroboration of delirium, we designed a prospective study to examine and characterize circulating leukocytes in patients with organ dysfunction undergoing heart surgery with CPB, who needed critical care. Furthermore, we aimed to develop and validate a nomogram for the accurate prediction of delirium in this study group.

## Materials and methods

### Study design and population

This prospective observational cohort study was performed with approval from the Institutional Review Board of Peking Union Medical College Hospital (PUMCH; approval number JS-1170). Written informed consent was obtained from patients’ legally authorized representatives. We included consecutive adult patients with organ dysfunction who were scheduled for elective or urgent cardiac surgery and required admission to the intensive care unit (ICU). Inclusion criteria were: (1) age ≥ 18 years; (2) admitted to the ICU for > 24 h; (3) screened positive for delirium based on the Richmond Agitation–Sedation Scale (RASS) and Confusion Assessment Method for the Intensive Care Unit (CAM-ICU); and (4) blood analyzed for candidate biomarkers at enrollment. Exclusion criteria were: (1) history of severe cognitive impairment or dementia resulting from hypoxic–ischemic encephalopathy, traumatic brain injury, cerebrovascular accident, intracranial infection, or brain death; (2) unable to speak or understand Mandarin; (3) any condition causing primary or acquired immunodeficiency within the previous 3 months, such as HIV infection, active autoimmune disease, hematological disease, or receiving chemotherapy or immunosuppressive drugs; and (4) refusal to participate.

### Procedures and data collection

#### Outcome measures

##### Identification of delirium

We used the confirmed simplified Chinese Version of CAM-ICU to assess all participants for delirium at admission and twice daily thereafter (Guenther et al., 2012; Wang et al., 2013). Delirium was confirmed if the RASS score was –3 to +4 and there was a positive CAM-ICU assessment. RASS and CAM-ICU have been validated in varied ICU populations and were used to detect delirium and coma throughout the ICU stay (Ely et al., 2001, 2003).

##### Delirium and delirium/coma-free days

To estimate duration of normal brain function free of delirium and coma, we used delirium/coma-free days as a surrogate of delirium duration. Delirium/coma-free days, reported in previous high-impact studies (Girard et al., 2018; van den Boogaard et al., 2018), were defined as the number of days a patient was alive and free from both delirium and coma after enrollment. Delirium onset and delirium/coma-free days were the primary outcomes of this study.

##### Other outcomes

Length of hospital and ICU stays, duration of mechanical ventilation, and 28-day mortality were recorded as secondary outcomes.

### Data collection

#### Demographics

Baseline demographic data were obtained at enrollment, including age, gender, comorbidity, and emergency admission. Acute Physiology and Chronic Health Evaluation II (APACHE II) and Sequential Organ Failure Assessment (SOFA) scores were recorded after the first 24 h of admission as an estimate of baseline severity of illness. Information about cardiac surgery was also collected: type of cardiac surgery, combined cardiac surgery (two or more surgeries during one operation), glucocorticoid treatment, CPB duration, and aortic cross-clamp (ACC) duration.

#### Clinical data

We carried out a thorough assessment of organ dysfunction, including vital signs, vasopressor medication, and indicators of cardiac, pulmonary, hepatic, renal and pancreatic function. Blood samples were collected at ICU admission for routine examination, including complete blood counts, high sensitivity C-reactive protein, procalcitonin, blood gas analysis, and biochemical blood tests.

#### Biomarkers and lymphocyte subsets

Peripheral blood samples were collected by PUMCH laboratories at enrollment with EDTA anticoagulant for measurement of lymphocyte counts and immunological biomarkers, as described previously (Li D. et al., 2020). Peripheral blood mononuclear cells were separated, stained with fluorescent monoclonal antibodies, and underwent flow cytometric analysis (3-color EPICSXL flow cytometer; Beckman Coulter, Brea, CA, United States) to detect T cells (CD3^+^), CD4^+^ T cells (CD4^+^CD3^+^ and CD28^+^CD4^+^), CD8^+^ T cells (CD8^+^CD3^+^, CD28^+^CD8^+^), B cells (CD19^+^), and natural killer cells (CD3^–^CD16^+^CD56^+^). (Supplementary Figure 1) Rate nephelometry (Array 360; Beckman Coulter) was used to measure serum levels of IgA, G, and M. Inflammatory markers were also recorded, including interleukin (IL)-6, IL-8, IL-10, and tumor necrosis factor (TNF)-α.

### Statistical analysis

Continuous data were presented as mean and standard deviation if normally distributed and analyzed using Student’s *t*-test. Otherwise, continuous data were presented as median and interquartile range (IQR) if not normally distributed and compared using the Mann–Whitney *U*-test. Categorical or numerical data were presented as numbers and percentages and compared using Pearson’s chi-square or Fisher’s exact test. Pearson’s correlation analysis was performed. Univariate analysis and multivariate linear regression analysis were used to examine the associations between delirium/coma-free days and variables related to delirium before and after adjustment for age, APACHE II score, SOFA score, length of ICU stay, duration of mechanical ventilation, and duration of physical restraint. Statistically significant variables were selected using conditional logistic regression with backward selection to identify risk factors associated with the onset of delirium. Receiver operating characteristic (ROC) curves and the area under the curves (AUCs) were examined in variables statistically significant associated with the onset of delirium, to determine a cut-off level and to predict development of delirium.

For nomogram development, all patients were randomly distributed into a training set and a validation set without replacement at a ratio of 7: 3 (247 from the training cohort and 132 from the validation cohort). Logistic regression analysis following the steps mentioned above was conducted to identify independent risk factors for the development of delirium (Supplementary Table 1). These independent predictors were used to prepare a predictive nomogram using the “rms” package. A nomogram for predicting the probability of delirium onset was obtained by the training set according to Occam’s Razor; namely, the best model should be one that can achieve the aim of study with the fewest variables (Van Den Berg, 2018). Individual predictors were marked with horizontal lines in the final nomogram. The area under the ROC curve (AUROC) was used to present the discriminative ability of the nomogram. Calibration of the nomogram was used for internal validation by the 1,000 bootstrap resampling procedure. Calibration curves were generated by plotting predicted vs. actual delirium rates to establish the accuracy of this nomogram. The clinical utility of the nomogram was assessed with the decision curve analysis (DCA) using the “rmda” package. For each patient, the total number of points based on the nomogram was calculated and the patients were stratified into high-, medium- and low-risk groups with a similar number of cases per group (Tang et al., 2019), to assess the association of the total score with delirium.

SPSS version 24.0 (IBM, Armonk, NY, United States), R for Windows version 4.12^1^ and GraphPad Prism for Windows version 9.0.0^2^ were used for the above statistical analysis. *P* < 0.05 in a two-tailed test was considered statistically significant.

## Results

### Baseline characteristics

**FIGURE 1:**
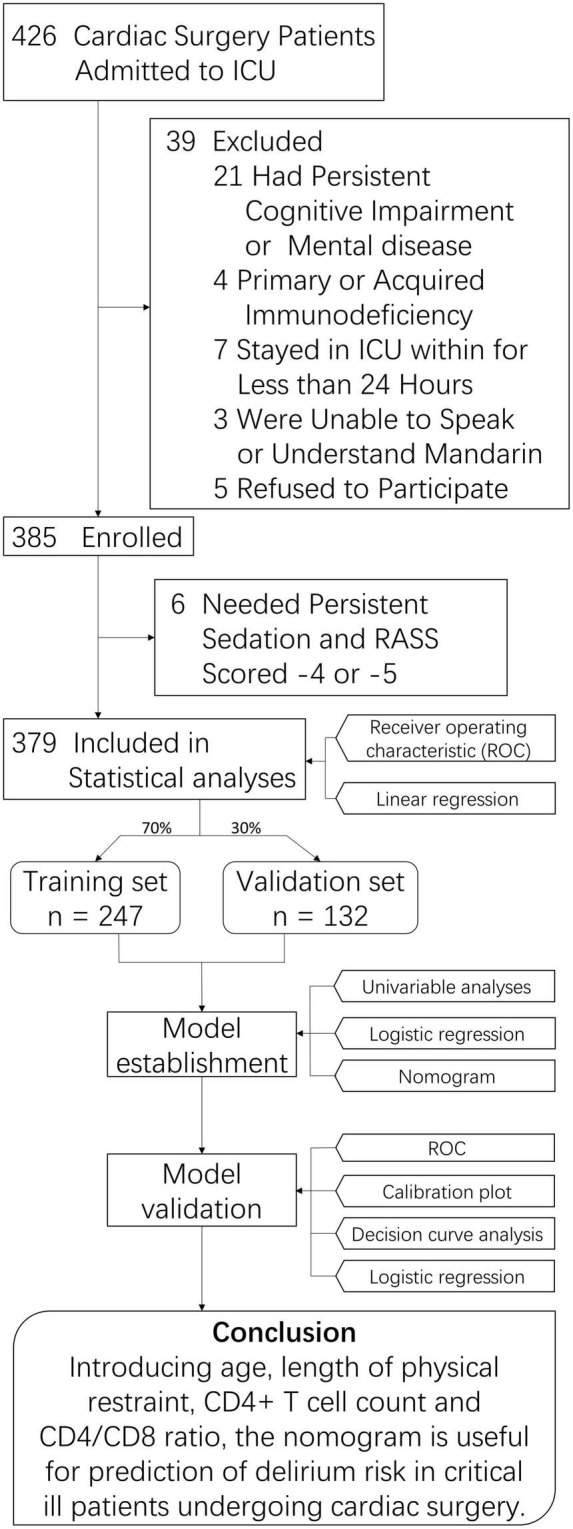
Study Flowchart. ICU, intessive care unit; CD4/CD8, CD4+ T cell count over CD8+ T cell count.

We selected 426 consecutive ICU patients undergoing cardiac surgery between January 2019 and August 2021. Thirty-nine were excluded, and 385 met the inclusion criteria. Six of the participants were later excluded because they had a constant need of sedation (RASS score –4 or –5). Therefore, 379 participants were included in the final analyses (Figure 1). All baseline characteristics are shown in Table 1. The participants were aged 55.34 ± 14.21 years (range 19–91 years) and were predominantly male (*n* = 250, 66.0%). The APACHE II score was 14.2 ± 5.6 and SOFA score was 18 ± 8.4 at ICU admission.

**TABLE 1:** Baseline characteristics of participants.

Characteristic	Total, *n* = 379, *n* (%)	Delirium, *n* = 242, *n* (%)	No delirium, *n* = 139, *n* (%)	*P-value*
Age, mean ± SD	55.3 ± 14.2	57.3 ± 13.5	51.8 ± 14.8	<0.001${}^{\underline{a}}$
Man	250 (66%)	158 (65.3%)	92 (66.2%)	0.859
**Comorbidity**				
Chronic cardiac insufficiency	281 (74.1%)	192 (79.3%)	89 (65.0%)	0.002${}^{\underline{a}}$
Chronic obstructive pulmonary disease	14 (3.7%)	9 (3.7%)	5 (3.6%)	0.973
Diabetes	88 (23.2%)	53 (21.9%)	35 (25.5%)	0.419
Chronic renal insufficiency	51 (13.5%)	40 (16.5%)	11 (8.0%)	0.020${}^{\underline{a}}$
Chronic hepatic insufficiency	7 (1.8%)	2 (0.8%)	5 (3.6%)	0.056
Solid tumor	13 (3.4%)	9 (3.7%)	4 (2.9%)	0.681
Immunologically mediated disease	13 (3.4%)	8 (3.3%)	5 (3.6%)	0.860
Blood diseases	13 (3.4%)	10 (4.1%)	3 (2.2%)	0.318
Emergency surgery	51 (13.5%)	36 (14.9%)	15 (10.9%)	0.282
SOFA score, mean ± SD	8.4 ± 5.4	9.0 ± 5.4	7.3 ± 5.3	0.003${}^{\underline{a}}$
APACHE II score, mean ± SD	14.2 ± 5.6	15.4 ± 5.7	12.1 ± 4.8	<0.001${}^{\underline{a}}$
**Vital Sign**				
Temperature, °C, mean ± SD	37.9 ± 0.8	37.9 ± 0.8	37.8 ± 0.7	0.999
Heart rate, bpm, mean ± SD	97.3 ± 14.4	98.2 ± 14.3	95.9 ± 14.6	0.140
Mean arterial pressure, mmHg, mean ± SD	83.5 ± 8.9	83.0 ± 9.1	84.3 ± 8.6	0.172
RR, bpm, mean ± SD	17.1 ± 3.6	17.3 ± 3.7	16.8 ± 3.5	0.203
Lactate, mmol/L, mean ± SD	2.7 ± 2.2	2.8 ± 2.3	2.7 ± 2.0	0.703
ScvO_2_,%, mean ± SD	73.2 ± 7.1	72.7 ± 7.3	74.1 ± 6.7	0.077
Pv-aCO_2_ gap, mmHg, mean ± SD	5.2 ± 2.1	5.4 ± 2.2	5.0 ± 2.0	0.165
No. of vasopressor medications, mean ± SD	1.4 ± 1.0	1.5 ± 0.9	1.3 ± 1.0	0.051
**Organ function**				
LVEF,%, mean ± SD	53.2 ± 6.9	52.8 ± 7.5	54.0 ± 5.8	0.116
PaO_2_/FiO_2_ Ratio, mmHg, mean ± SD	323.1 ± 136.9	301.7 ± 123.2	360.9 ± 151.5	<0.001${}^{\underline{a}}$
Serum creatinine, μmol/L, mean ± SD	108.3 ± 91.2	119.2 ± 99.5	89.1 ± 70.7	0.001${}^{\underline{a}}$
Blood urea nitrogen, μmol/L, mean ± SD	8.4 ± 6.1	9.1 ± 6.3	7.1 ± 5.4	0.002${}^{\underline{a}}$
ALT, U/L, mean ± SD	39.0 ± 140.9	37.8 ± 103.0	41.1 ± 190.6	0.821
Total bilirubin, μmol/L, mean ± SD	23.2 ± 15.5	24.7 ± 17.2	20.5 ± 11.5	0.012${}^{\underline{a}}$
Direct bilirubin, μmol/L, mean ± SD	8.8 ± 10.7	9.8 ± 12.4	7.1 ± 6.5	0.006${}^{\underline{a}}$
Serum amylase, μ/L, mean ± SD	111.6 ± 201.2	119.3 ± 200.9	98.1 ± 201.8	0.327

${}^{\underline{a}}$Statistically significant (P < 0.05).

SD, standard deviation; SOFA, Sequential Organ Failure Assessment; APACHE II, Acute Physiology and Chronic Health Evaluation II; ScvO_2_, central venous oxygen saturation; Pv-aCO_2_ gap, veno-arterial difference in the partial pressure of carbon dioxide; LVEF, left ventricular ejection fraction; ALT, alanine aminotransferase.

### Incidence of delirium and related variables

Delirium developed in 242 of the 379 participants during the 2124 observational days in ICU, leading to an incidence rate of 63.9% [95% confidence interval (CI) 59.0–68.7%]. Patients with chronic cardiac insufficiency were more likely to develop delirium (79.3% vs. 65.5% in those without chronic cardiac insufficiency, *p* = 0.003). There were some significant differences between the two study groups in terms of organ function: SOFA score (9.0 ± 5.4 vs. 7.3 ± 5.3 in those without delirium, *p* = 0.003), APACHE II score (15.4 ± 5.7 vs. 12.1 ± 4.8 in those without delirium, *p* < 0.001), PaO_2_/FiO_2_ ratio (301.7 ± 123.2 vs. 360.9 ± 151.5, *p* < 0.001), serum creatinine (119.2 ± 99.5 vs. 89.1 ± 70.7, *p* = 0.001), blood urea nitrogen (9.1 ± 6.3 vs. 7.1 ± 5.42, *p* = 0.002), total bilirubin (24.7 ± 17.2 vs. 20. 5 ± 11.5, *p* = 0.012), and direct bilirubin (9.8 ± 12.4 vs. 7.1 ± 5.6, *p* = 0.006). The characteristics of the study population are shown in Table 1.

**FIGURE 2:**
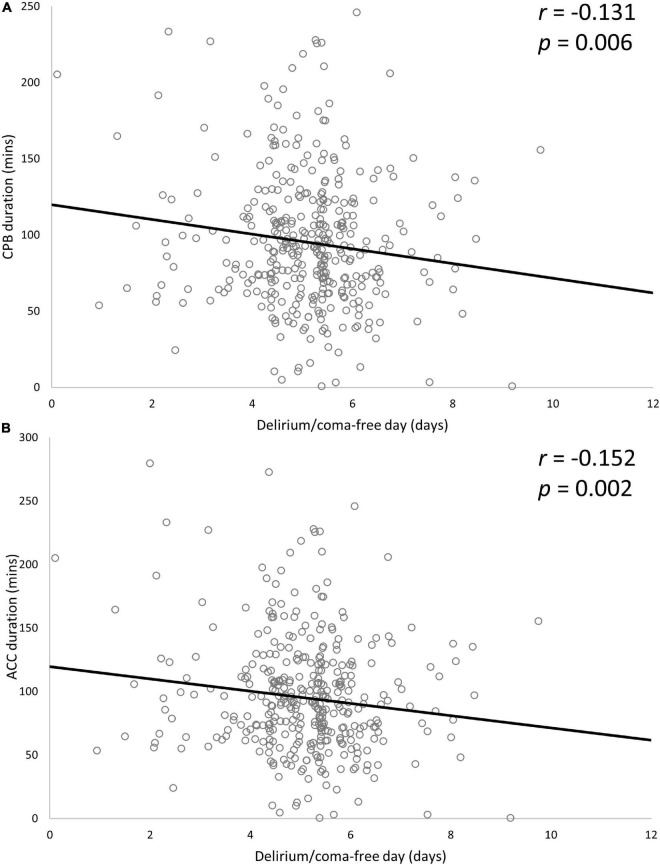
Associations between delirium/coma-free day and CPB duration **(A)** and ACC duration **(B)**. Two variables were significantly negatively associated with delirium/coma-free days. Partial r and *p*-values were obtained after adjustment for age, APACHE II score, SOFA score, length of ICU stays, duration of mechanical ventilator and duration of physical restraints. CPB, cardiopulmonary bypass; ACC, aortic cross-clamp; SOFA, Sequential Organ Failure Assessment; APACHE II, Acute Physiology and Chronic Health Evaluation II; ICU, intensive care unit.

Patients who had coronary artery bypass graft (CABG) had a higher risk of developing delirium (38.8% vs. 27.0% in those without CABG; *p* = 0.027), but no differences were identified for other types of surgery or combined surgery. Patients with delirium had longer CBP duration (149.6 ± 59.1 vs. 126.7 ± 48.5 min, *p* < 0.001) and ACC duration (98.7 ± 51.5 vs. 86.1 ± 41.6 min, *p* = 0.010) than those without delirium. There was no significant difference in whether to use intraoperative glucocorticoids (0.8% vs. 2.2% in those without delirium, *p* = 0.276). Patients with delirium were more likely than those without delirium to receive midazolam (90.5% vs. 70.8%, *p* < 0.001) and dexmedetomidine (45.0% vs. 10.9%, *p* < 0.001). There was a significant difference between the delirium and non-delirium groups for physical restraint (100% vs. 98.5%, *p* = 0.043) and duration of physical restraint (135.2 ± 7.5 vs. 32.7 ± 34.3 h, *p* < 0.001). Patients with delirium had prolonged duration of mechanical ventilation (99.97 ± 103.37 vs. 29.52 ± 20.94 h, *p* < 0.001), median length of ICU stay (5.6 days, IQR 5 days vs. 2.0 days, IQR 1.9 days; *p* < 0.001) and median length of hospital stay (23 days, IQR 14 days vs. 18 days, IQR 8 days; *p* < 0.001) compared with patients without delirium. The variables related to the perioperative period are shown in Table 2. Duration of CPB and ACC were significantly negatively associated with delirium/coma-free days for patients with delirium (CPB time: partial *r* = −0.131, *p* = 0.006; ACC time: partial *r* = − 0.152, *p* = 0.002) (Figure 2).

**TABLE 2:** Variables in the perioperative period.

Variable	Total, *n* (%)	Delirium, *n* (%)	No delirium, *n* (%)	*P-value*
Types of cardiac surgery				
CABG	131 (34.6%)	94 (38.8%)	37 (27.0%)	0.027${}^{\underline{a}}$
Valve surgeries	185 (48.8%)	111 (45.9%)	74 (54.0%)	0.156
Cardiac tumor resection	28 (7.4%)	13 (5.4%)	15 (10.9%)	0.073
Aortic arch repairs	41 (10.8%)	29 (12.0%)	12 (8.8%)	0.424
Others	23 (6.1%)	17 (7.0%)	6 (4.4%)	0.417
Combined cardiac surgeries	27 (7.1%)	21 (8.7%)	6 (4.4%)	0.175
Glucocorticoid treatment	5 (1.3%)	2 (0.8%)	3 (2.2%)	0.276
CPB duration, min, mean (SD)	141.4 (56.5)	149.6 (59.1)	126.8 (48.5)	<0.001${}^{\underline{a}}$
ACC duration, min, mean (SD)	94.2 (48.5)	98.7 (51.5)	86.1 (41.6)	0.010${}^{\underline{a}}$
Use of propofol	363 (95.8%)	232 (95.9%)	131 (95.6%)	1.000
Use of midazolam	316 (83.4%)	219 (90.5%)	97 (70.8%)	<0.001${}^{\underline{a}}$
Use of dexmedetomidine	124 (32.7%)	109 (45.0%)	15(10.9%)	<0.001${}^{\underline{a}}$
Sepsis	66 (17.4%)	46 (19.0%)	20 (14.6%)	0.344
Physical restraint	377 (99.5%)	242 (100%)	135 (98.5%)	0.043${}^{\underline{a}}$
Duration of physical restraint, mean (SD)	97.7 (115.4)	135.2 (127.8)	31.3 (33.2)	<0.001${}^{\underline{a}}$
Outcomes				
Length of ICU stay, days, Mdn (IQR)	5.6 (5.5)	7.2 (6.2)	2.8 (1.7)	<0.001${}^{\underline{a}}$
Length of hospital stay, days, Mdn (IQR)	24.6 (14.2)	26.9 (15.9)	20.6 (9.2)	<0.001${}^{\underline{a}}$
Duration of mechanical ventilator, hours, mean (SD)	74.5 (90.1)	99.97 (103.37)	29.52 (20.94)	<0.001${}^{\underline{a}}$
28-day mortality	11 (2.9%)	8 (3.3%)	3 (2.2%)	0.525

${}^{\underline{a}}$Statistically significant (P < 0.05).

CABG, coronary artery bypass graft; SD, standard deviation; CPB, cardiopulmonary bypass; ACC, aortic cross-clamp; ICU, intensive care unit; IQR, interquartile range.

### Comparison of immune parameters between delirium and non-delirium groups

The following inflammatory markers were higher in the delirium than non-delirium group: IL-6 (median 247.2 pg/mL, IQR 383.3 pg/mL vs. median 178.0 pg/mL, IQR 274.1 pg/mL, *p* = 0.009); IL-8 (median 101.0 pg/mL, IQR 108.3 pg/mL vs. median 74.0 pg/mL, IQR 97.0 pg/mL, *p* = 0.029); and TNF-α (median 12.3 pg/mL, IQR 6.2 pg/mL vs. median 8.9 pg/mL, IQR 5.6 pg/mL, *p* < 0.001). There were no significant differences between the groups for other inflammatory markers (IL-10, neutrophil count, lymphocyte count, neutrophil to lymphocyte ratio, high sensitivity C-reactive protein, and procalcitonin or immune parameters (IgA, G, and M, and complement components 3 and 4). Some lymphocyte subsets were significantly higher in the delirium than non-delirium group: CD19^+^ B cell count (median 145.5 cells/μL, IQR 160 cells/μL vs. median 105 cells/μL, IQR 111 cells/μL, *p* = 0.004); and CD4^+^ T cell count (median 566 cells/μL, IQR 182 cells/μL vs. median 339 cells/μL, IQR 253 cells/μL, *p* < 0.001). There were no significant differences in natural killer cell count, CD3^+^ T cell count or CD8^+^ T cell count. There was a significant difference in CD4/CD8 ratio (median 2.31, IQR 1.00 in delirium group vs. median 1.28, IQR 0.83 in non-delirium group, *p* < 0.001). The variables of inflammatory and immune parameters are shown in Table 3.

**TABLE 3:** Inflammatory and immune markers in critically ill patients undergoing cardiac surgery.

Variable	Total, *n* (%)	Delirium, *n* (%)	No delirium, *n* (%)	*P-value*
Inflammatory markers				
Interleukin-6, pg/mL, Mdn (IQR)	208.7 (346.7)	247.2 (383.3)	178.0 (274.1)	0.009${}^{\underline{a}}$
Interleukin-8, pg/mL, Mdn (IQR)	89.0 (105.0)	101.0 (108.3)	74.0 (97.0)	0.029${}^{\underline{a}}$
Interleukin-10, pg/mL, Mdn (IQR)	9.9 (10.6)	10.0 (10.8)	9.6 (10.1)	0.668
Tumor necrosis factor-α, pg/mL, Mdn (IQR)	11.3 (6.9)	12.3 (6.2)	8.9 (5.6)	<0.001${}^{\underline{a}}$
Neutrophil count, × 10^9/L, mean (SD)	11.74 (4.99)	12.01 (5.27)	11.27 (4.45)	0.143
Lymphocyte count, × 10^9/L, mean (SD)	1.16 (0.43)	1.18 (0.43)	1.12 (0.43)	0.143
NLR, Mdn (IQR)	10.17 (5.60)	10.23 (5.64)	10.00 (5.39)	0.698
hsCRP, mg/dL, Mdn (IQR)	121.8 (152.06)	140.76 (163.68)	113.95 (113.14)	0.094
PCT, ng/L, mean (SD)	10.64 (29.07)	10.72 (18.26)	10.50 (42.00)	0.943
Immune parameters, g/L, mean (SD)				
Immunoglobulin A	1.91 (0.77)	1.90 (0.76)	1.92 (0.78)	0.751
Immunoglobulin G	8.52 (3.02)	8.33 (2.92)	8.84 (3.20)	0.122
Immunoglobulin M	0.72 (0.45)	0.71 (0.44)	0.74 (0.47)	0.602
Complement 3	0.773 (0.217)	0.760 (0.234)	0.796 (0.181)	0.097
Complement 4	0.157 (0.056)	0.155 (0.059)	0.160 (0.052)	0.361
Lymphocyte subsets, Mdn (IQR)				
Natural killer cell count, cells/μL	92 (89)	94 (84)	86 (95)	0.211
CD19 + B cell count, cells/μL	129 (142)	145.5 (160)	105 (111)	0.004${}^{\underline{a}}$
CD3 + T cell count, cells/μl	827 (328)	844.5 (281)	809 (463)	0.058
CD4 + T cell count, cells/μl	528 (301)	566 (182)	339 (253)	<0.001${}^{\underline{a}}$
CD8 + T cell count, cells/μl	251 (147)	251.5 (134)	244 (207)	0.690
CD4/CD8 ratio	1.93 (1.16)	2.31 (1.00)	1.28 (0.83)	<0.001${}^{\underline{a}}$

${}^{\underline{a}}$Statistically significant (P < 0.05).

IQR, interquartile range; hsCRP, hypersensitive C-reactive protein; PCT, procalcitonin; NLR, neutrophil to lymphocyte ratio; SD, standard deviation.

**FIGURE 3:**
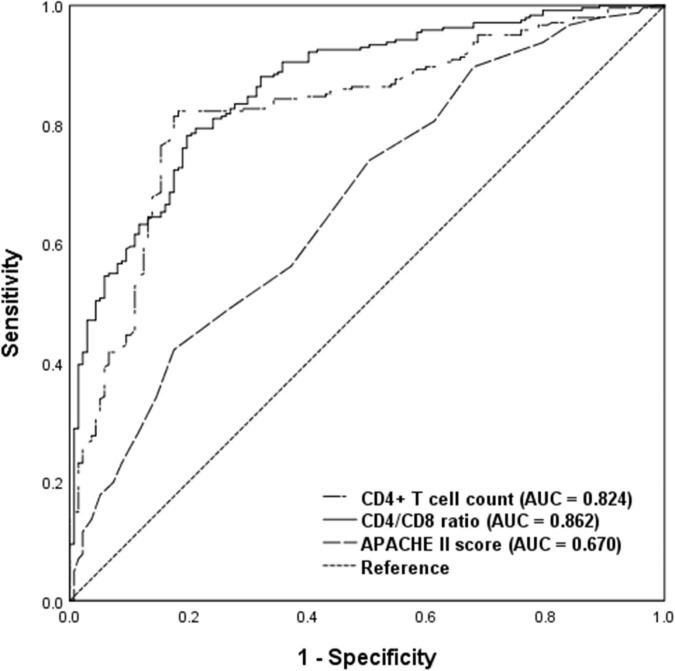
Receiver operating characteristic analysis of immune parameters predicting delirium among critical care patients undergoing cardiac surgery. AUC, area under curve; APACHE II, acute physiology and chronic health evaluation II.

To evaluate the discriminatory ability for predicting development of delirium, ROC analysis was applied to the immune parameters that differed significantly between the delirium and non-delirium groups. Compared with APACHE II score, CD4^+^ T cell count and CD4/CD8 ratio had the greatest discriminatory ability. AUC was 0.824 (95% CI: 0.780–0.868) for CD4^+^ T cell count and 0.862 (95% CI: 0.825–0.90) for CD4/CD8 ratio, compared with 0.670 (95% CI: 0.614–0.726) for APACHE II score (Figure 3).

### Risk factors for delirium

Based on univariate analyses, 18 significant variables were included in the binary multivariate logistic regression model: age, chronic cardiac insufficiency, SOFA score, APACHE II score, PaO_2_/FiO_2_ ratio, serum creatinine, total bilirubin, CABG, CPB duration, use of midazolam, duration of physical restraint, length of hospital stay, IL-6, IL-8, TNF-α, CD19^+^ B cell count, CD4^+^ T cell count, and CD4/CD8 ratio. Binary logistic regression analysis identified six independent risk factors for delirium among critically ill patients undergoing cardiac surgery: age (OR 1.030, 95% CI 1.002–1.058, *p* = 0.038); duration of physical restraint (OR 1.030, 95% CI 1.019–1.040, *p* < 0.001); IL-6 (OR 1.001, 95% CI 1.00–1.003, *p* = 0.025); CD19^+^ B cell count (OR 0.996, 95% CI 0.993–0.999, *p* = 0.016); CD4^+^ T cell count (OR 1.005, 95% CI 1.003–1.008, *p* < 0.001); and CD4/CD8 ratio (OR 5.314, 95% CI 2.796–10.099, *p* < 0.001) (Table 4).

**TABLE 4:** Logistic regression analysis of risk factors associated with delirium.

	B	SE	*P*-value	OR	95% CI	
Age	0.029	0.014	0.038${}^{\underline{a}}$	1.030	1.002	1.058
Length of physical restraint	0.029	0.005	<0.001${}^{\underline{a}}$	1.030	1.019	1.040
Interleukin-6	0.001	0.001	0.025${}^{\underline{a}}$	1.001	1.000	1.003
CD19 + B cell count	–0.004	0.002	0.016${}^{\underline{a}}$	0.996	0.993	0.999
CD4 + T cell count	0.005	0.001	<0.001${}^{\underline{a}}$	1.005	1.003	1.008
CD4/CD8 ratio	1.670	0.328	<0.001${}^{\underline{a}}$	5.314	2.796	10.099

${}^{\underline{a}}$Statistically significant (P < 0.05).

SE, Standard Error; OR, odds ratio; 95% CI, 95% confidence interval.

### Nomogram model for predicting risk of delirium

**FIGURE 4:**
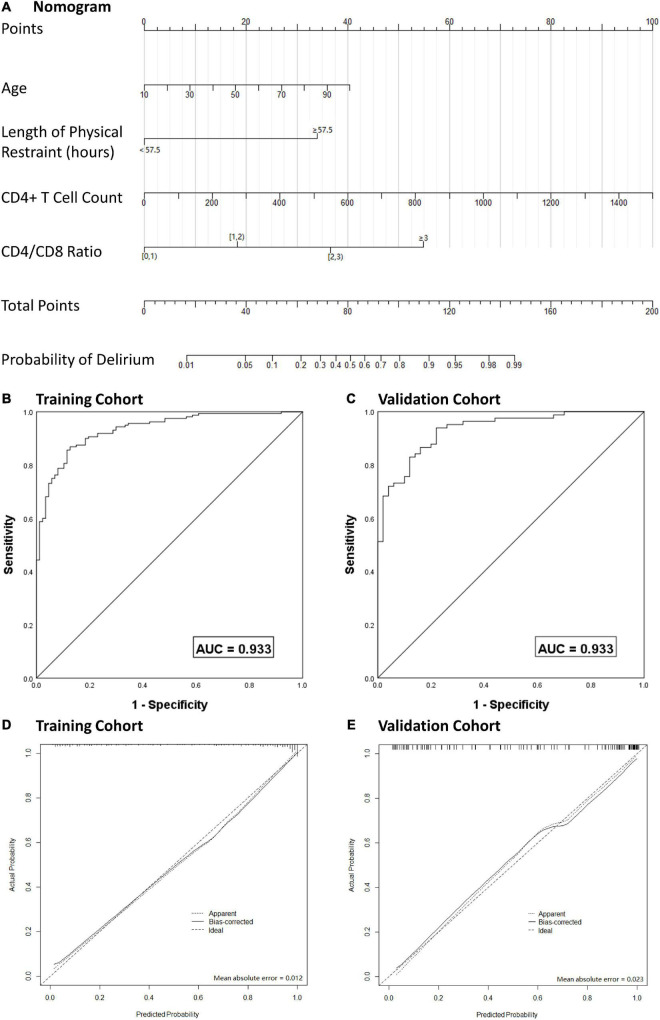
Nomogram model for the prediction of individual delirium risk in critical ill patients undergoing cardiac surgery, **(A)** nomogram for delirium risk. A comparison of area under the receiver operating characteristic curve (AUROC) values for the prediction of delirium in the training cohort **(B)** and validation cohort **(C)**. Calibration curves for nomogram-based assessments of the training cohort **(D)** and validation cohort **(E)**. CD4/CD8, CD4 + T cell count over CD8 + T cell count; AUC, area under curve.

After multivariate logistic regression analysis, the independent predictors including age, CPB duration, CD4^+^ T cell count and CD4/CD8 ratio (Table 4) were used to develop a nomogram. The score of each variable in the nomogram was obtained by drawing a vertical line upward to the score scale line at the top. The total score of the patient was obtained by adding the scores of all variables, and the predictive probability of delirium occurrence was obtained by drawing a vertical line downward to the predictive probability line through the total score line at the bottom. For example, for a 60-year-old ICU patient after cardiac surgery, with CPB duration 150 min, CD4^+^ T cell count 700/μL and CD4/CD8 ratio 2.0, the total score was 94 points. This corresponded to a risk of delirium after cardiac surgery of ∼0.85. The ROC curve was used to analyze the discriminatory degree of the nomogram model. ROC analyses revealed the AUROC for this nomogram in the training and validation cohorts to be 0.919 and 0.883, respectively (Figure 4).

To prevent overfitting of the model, the bootstrap resampling method was used for internal verification and deviation correction. The bootstrap resampling process was repeated 1000 times to obtain the calibration curve after deviation correction. The calibration curve showed that the mean absolute error between prediction of risk of delirium development by the nomogram model and actual delirium risk was 0.017, which indicated that the nomogram model had good discrimination and calibration.

**FIGURE 5:**
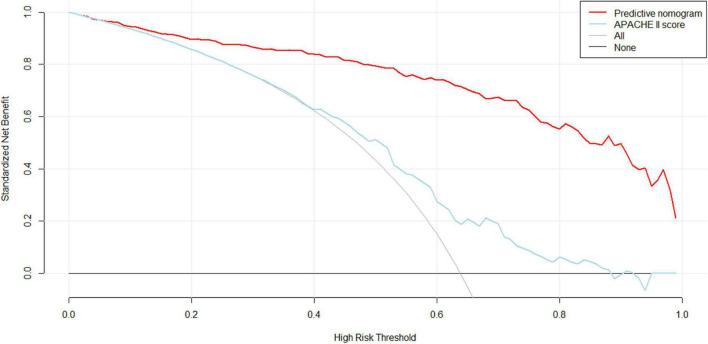
Decision curve analysis (DCA) of the nomogram. X-axis and y-axis represent threshold probability and net benefit, respectively. For the clinical utility of the nomogram as a diagnostic model for delirium, the net benefit curve is shown in the DCA. When the threshold value of the diagnostic model is between 0.30 and 0.99, the patients will obtain the corresponding net benefit as long as the therapeutic measure is taken.

With regard to clinical use, DCA for the nomogram was carried out. The threshold probability of the nomogram ranged from 30 to 99%, which suggested a wide range of clinical utility. As long as the predictive probability for ICU patients undergoing cardiac surgery with delirium was 30–99%, the nomogram delivered the standard net benefit in varying degrees under the corresponding intervention measure. The clinical utility of the nomogram to predict delirium is shown in Figure 5.

**FIGURE 6:**
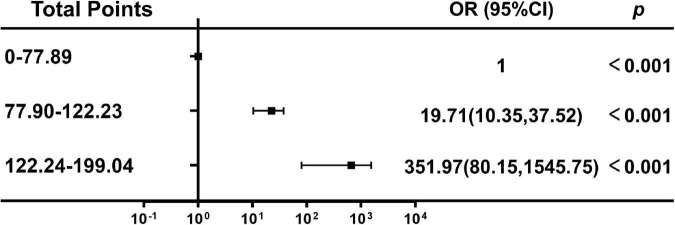
Association between the total points of the nomogram and the risk of delirium. OR, odds ratio; Cl, confidence interval.

According to nomogram-predicted risk scores, patients from the overall cohort were classified into high-, moderate- and low-risk groups. Risk of delirium increased with total points. The moderate-risk group (total points: 70.13–92.04, OR 19.71, 95% CI 10.35–37.52, *p* < 0.001) and high-risk group (total points: 122.24–199.04, OR 351.97, 95% CI 80.15–1545.75, *p* < 0.001) had a higher delirium onset risk than the low-risk group had (total points: 0–70.12) (Figure 6).

## Discussion

To our knowledge, this is the first study to show that dysregulated immune response, measured by peripheral blood lymphocyte subsets, was independently associated with and had a predictive value for delirium in critically ill patients undergoing cardiac surgery, and to develop a nomogram for accurate prediction of delirium in this cohort. We focused on peripheral blood lymphocyte subsets and inflammatory markers to further characterize patients who have already developed delirium; a ubiquitous complication in the ICU (Khan et al., 2020). In cardiac surgery patients with organ dysfunction, who required critical care, we observed the following. (1) High CD4^+^ T cell count and CD4/CD8 ratio were significantly associated with a high incidence of developing delirium, and ROC analysis confirmed their predictive performance. (2) Decreased CD19^+^ B cell count, increased CD4^+^ T cell count and CD4/CD8 ratio, elevated IL-6, and advanced age were independent risk factors associated with delirium onset. (3) The nomogram predicted that delirium was associated with age, duration of CPB, CD4 T cell count and CD4/CD8 ratio.

In cardiac surgery, CPB is associated with a systemic inflammatory response syndrome caused by release of proinflammatory mediators, primarily cytokines and reactive oxygen species (Bronicki and Bleiweis, 2014). Delirium is a common complication in cardiac surgery patients (Sauër et al., 2017; Greaves et al., 2020). The finding that duration of CPB was associated with delirium could represent worse underlying disease, more complex surgery, and harder perioperative care. Hence, we investigated the correlation between duration of CPB and the occurrence and duration of delirium. The results were consistent with our hypothesis that duration of CPB was independently associated with delirium development, which is in line with previous work (Guenther et al., 2013). We found that duration of CPB and ACC was significantly negatively associated with delirium/coma-free days, which means that patients with longer duration of CPB and ACC had prolonged delirium or coma. We successfully fitted duration of CPB into the nomogram for predicting delirium, which could give clinicians a more accurate way to predict and prevent delirium. In cardiac surgery, reduced cerebral perfusion may play an important role in the pathogenesis of delirium because extremes of blood pressure are common (Vedel et al., 2018). By using cerebral oxygen monitoring and near-infrared spectroscopy, researchers have identified that impaired cerebral autoregulation occurs frequently in the operating theater and ICU for cardiac surgery patients (Nakano et al., 2021). Some studies have reported reduced incidence of delirium by maintaining the mean arterial pressure above the lower limit of brain autoregulation (Siepe et al., 2011; Brown et al., 2019).

Our results indicate the role of cellular immunity and neuroinflammation in the development of delirium, and provide evidence that assessment of lymphocyte subsets, especially CD4^+^ T cells and CD4/CD8 ratio, is important for understanding this process. Activated peripheral T cells express adhesion molecules and chemokine receptors that enable them to cross the blood–brain barrier (BBB) and infiltrate brain tissue (Ransohoff and Engelhardt, 2012; Sambucci et al., 2019). The hyper-responsiveness of brain immune cells to stimulation from peripheral inflammation makes the brain susceptible to the consequences of systemic inflammation (Mariscalco et al., 2015), which leads to cognitive impairment and delirium (Simone and Tan, 2011). CD4^+^ T cells play a central role in the body’s defense system because of their impact on the function of B cells and cells in the innate immune system (Wu et al., 2012). Based on their unique extracellular cytokine milieu and main regulatory function, CD4^+^ T cells can be further classified into at least four functionally distinct subsets: T helper (Th)1, Th2, Th17, and regulatory T cells (Street and Mosmann, 1991; Bettelli et al., 2006; Wu et al., 2012). Our results are consistent with and extend those of recently published studies. An increase in CD4^+^ T cells and decrease in CD8^+^ T cells have been observed in patients with Alzheimer’s disease (Richartz-Salzburger et al., 2007). In a model of traumatic brain injury, mice treated with an immunomodulatory agent that inhibited T cell trafficking to the central nervous system (CNS) had decreased T and NK cell infiltration, as well as improved neurological function, brain edema and BBB damage (Gao et al., 2017). The development of acute multiple sclerosis and its animal model shows that Th17 cells migrate from the periphery, cross the BBB and accumulate in the CNS (Brucklacher-Waldert et al., 2009; Kebir et al., 2009), injuring neurons and promoting neuroinflammation through CD4^+^ T cell recruitment (Kebir et al., 2007; Cipollini et al., 2019).

To further understand the role that inflammation plays in delirium, we measured levels of inflammatory markers, and found that increased IL-6 and TNF-α were significantly associated with delirium development. Delirium has been associated with increased proinflammatory circulating IL-6 in ischemic stroke patients (Kowalska et al., 2018) and in patients after open heart surgery (Plaschke et al., 2010). IL-6 is a regulator of Th17 differentiation *in vitro* and a potential target for inhibition of Th17 development *in vivo* (Ji et al., 2019). It is an important mediator in various immunological and inflammatory processes that play a role in the pathogenesis of CNS disorders. Elevated levels of IL-6 and TNF-α compromise BBB integrity though nicotinamide adenine dinucleotide phosphate oxidase activation and reactive oxygen species generation (Rochfort and Cummins, 2015). We suppose that delirium is associated with increased proinflammatory cytokines (such as IL-6 and TNF-α), which leads to inappropriate activation of peripheral CD4^+^ T cells and exacerbation of neuroinflammation in the brain, with BBB disruption.

To date, there is no model for predicting the occurrence of delirium in critically ill patients undergoing cardiac surgery. The optimal predictive model was represented by a visualization nomogram. The nomogram was a reliable tool for clinical decision-makers to predict the development of delirium. We established a predictive model and incorporated the following four variables into its construction: age, duration of physical restraint, CD4^+^ T cell count, and CD4/CD8 ratio. Our clinical prediction nomogram had face validity in that age and duration of physical restraint have been identified in previous studies (Li X. et al., 2020; Dong et al., 2021). Importantly, the identification of the additional risk factors of CD4^+^ T cell count and CD4/CD8 ratio extended the previous results. Validation of the model using different statistical methods demonstrated its performance. The AUROC was high for this nomogram in the training and validation cohorts. The calibration curves also showed good consistency for the probability of delirium between the two cohorts. DCA exhibited positive net benefits in the predictive model, indicating the favorable potential clinical effect of the predictive model. In addition, the forest plot (Figure 6) showed that the model has good predictive ability and clinical application potential. For application of this model, the predictive nomogram is therefore essential to an improved risk stratification process in critically ill patients undergoing cardiac surgery, and can be used to screen out patients with a high risk of developing delirium. Simultaneously, it proved that these two immune-related factors (CD4^+^ T cell count and CD4/CD8 ratio) were valuable to understand the pathophysiology of delirium. Therefore, targeted interventions can be made for high-risk patients.

APACHE II and SOFA scores represent illness severity and organ dysfunction, respectively. The APACHE II and SOFA scores in our study were 14.2 ± 5.7 and 8.4 ± 5.4, respectively, indicating severe illness in our patients and explaining the high incidence of delirium, which is supported by other studies (Rahimi-Bashar et al., 2021). We found that delirium was a frequent complication in older ICU patients, which was similar to previous studies (Tafelmeier et al., 2019; Eertmans et al., 2020). Aging is an inevitable trend worldwide (Sun et al., 2021), and could cause an increase in IL-6 and TNF-α in the CNS and peripheral bloodstream even in the absence of disease (Krabbe et al., 2004). The aged brain is more fragile to neuroinflammation because of cellular senescence resulting from senescence-associated secretory phenotype and immunosenescence (Guerrero et al., 2021). Acute elevation of TNF-α has robust effects on brain function in the degenerating brain (Hennessy et al., 2017). Besides, the transcriptional age of brain endothelial cells, key constituents of the BBB, is sensitive to age-related circulatory cues (Chen et al., 2020). Together, these results demonstrate that advanced age is an independent risk factor and has predictive value for delirium. Our results showed that high serum creatinine and blood urea nitrogen in patients with delirium were commensurate with comorbidity of chronic renal insufficiency, and indicate renal hypoperfusion during the perioperative period. Animal studies have suggested that experimental renal ischemia can lead to a vigorous neuroinflammatory response, brain dysfunction, and BBB disruption, with increased cytokines including IL-6 and TNF-α (Liu et al., 2008). The results of this study implied that delirium patients used sedatives more often than other patients, particularly midazolam and dexmedetomidine, which highlighted the importance of minimal sedation (Vincent et al., 2016) and sequential sedation (Yang et al., 2017).

### Study limitations

Several limitations need to be discussed. First, owing to the difficulties of obtaining cerebrospinal fluid (CSF) from critically ill patients, especially those with unstable hemodynamics after cardiac surgery, we did not collect CSF from our patients. This meant that we could not assess lymphocyte subsets and inflammatory markers in the CSF and further elucidate the changes within the CNS. Second, our study did not dynamically monitor the changes in lymphocyte subsets and inflammatory markers during delirium. So, the relationships between delirium duration and dynamic changes in peripheral blood lymphocyte subsets and inflammatory markers were not detected. Third, the differentiation of CD4^+^ T cells, such as Th1, Th17 and regulatory T cells, their transcription factors and secreted effector molecules (such as STAT1, STAT3, IFNγ, and IL-17) were not investigated, which limited the depth of our study. Further studies should be conducted to take account of these limitations.

## Conclusion

We identified predictive risk factors for delirium in critically ill patients undergoing cardiac surgery, and a novel nomogram was developed and validated. Our findings highlighted the importance of biomarkers of inflammation and inappropriate CD4^+^ T cell activation in the onset of delirium. This suggests a potential role for CD4^+^ T cells in mediating neuroinflammation in delirium and providing potential biomarkers for delirium.

## Data availability statement

The original contributions presented in this study are included in the article/Supplementary material, further inquiries can be directed to the corresponding author.

## Ethics statement

The studies involving human participants were reviewed and approved by the Institutional Review Board of Peking Union Medical College Hospital (PUMCH; approval number: JS-1170). The patients/participants provided their written informed consent to participate in this study.

## Author contributions

WC and DL collected and initially screened the data. NC, JZ, and FW guided the research ideas of the full text. XL performed a visual analysis of the data and was the main contributor to the manuscript. All authors contributed to the article, approved the submitted version, and qualify as per ICJME criteria for authorship.

## Footnotes

1
http://www.r-project.org/

2
http://www.graphpad.com/

## Supplementary material

Supplementary Figure 1 Gating strategies of lymphocyte subsets for the present study.

Supplementary Table 1 Comparison of patients between the training and validation sets.
