# Supplementary figures and images for: Early alteration of peripheral blood lymphocyte subsets as a risk factor for delirium in critically ill patients after cardiac surgery: A prospective observational study
Source: Front Aging Neurosci. 2022 Sep 1;14:950188. doi: 10.3389/fnagi.2022.950188 (PMC9477480; doi:10.3389/fnagi.2022.950188)

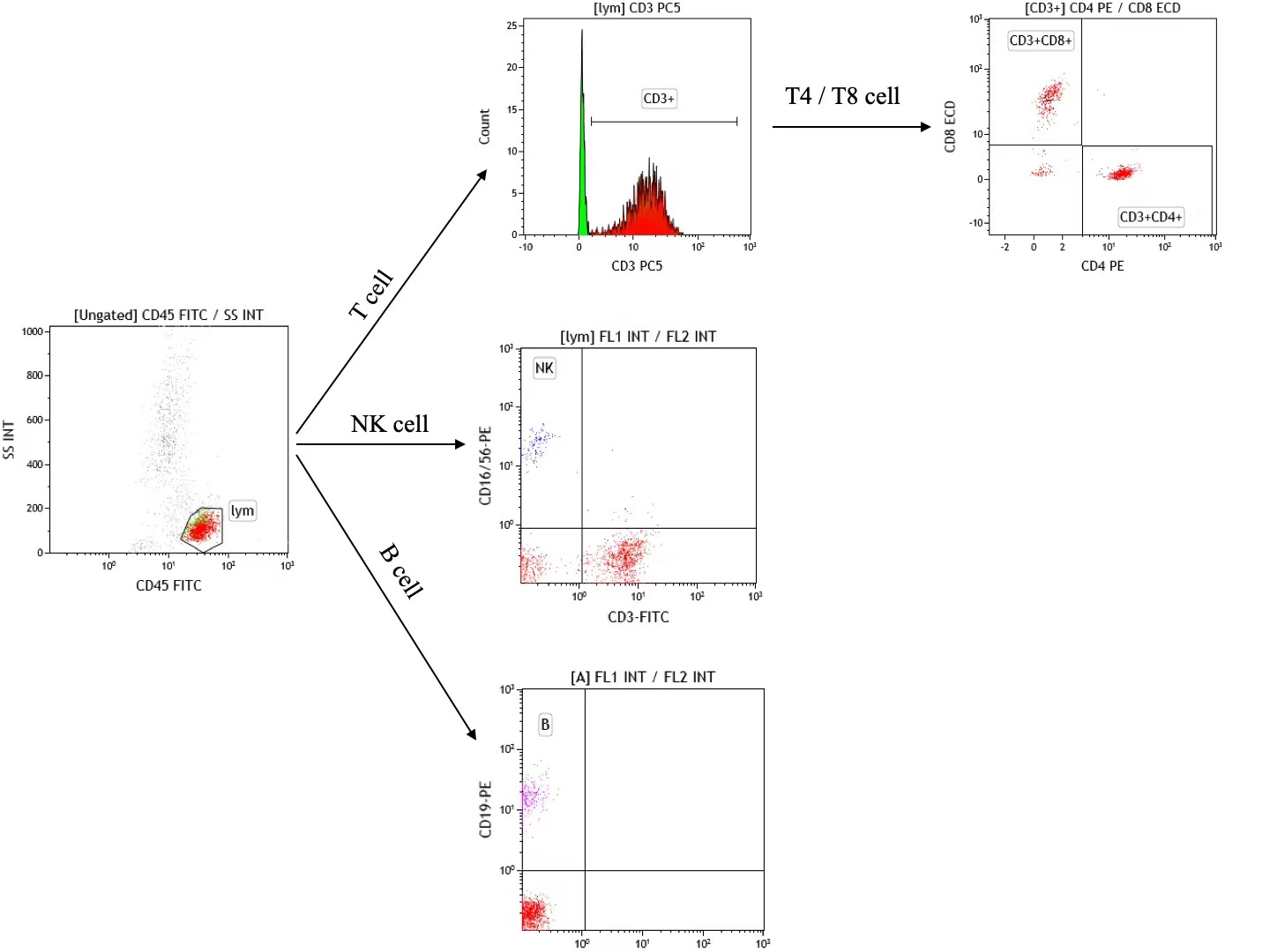

Supplement: Supplementary Figure 1 — Gating strategies of lymphocyte subsets for the present study. [file Image_1.TIF]

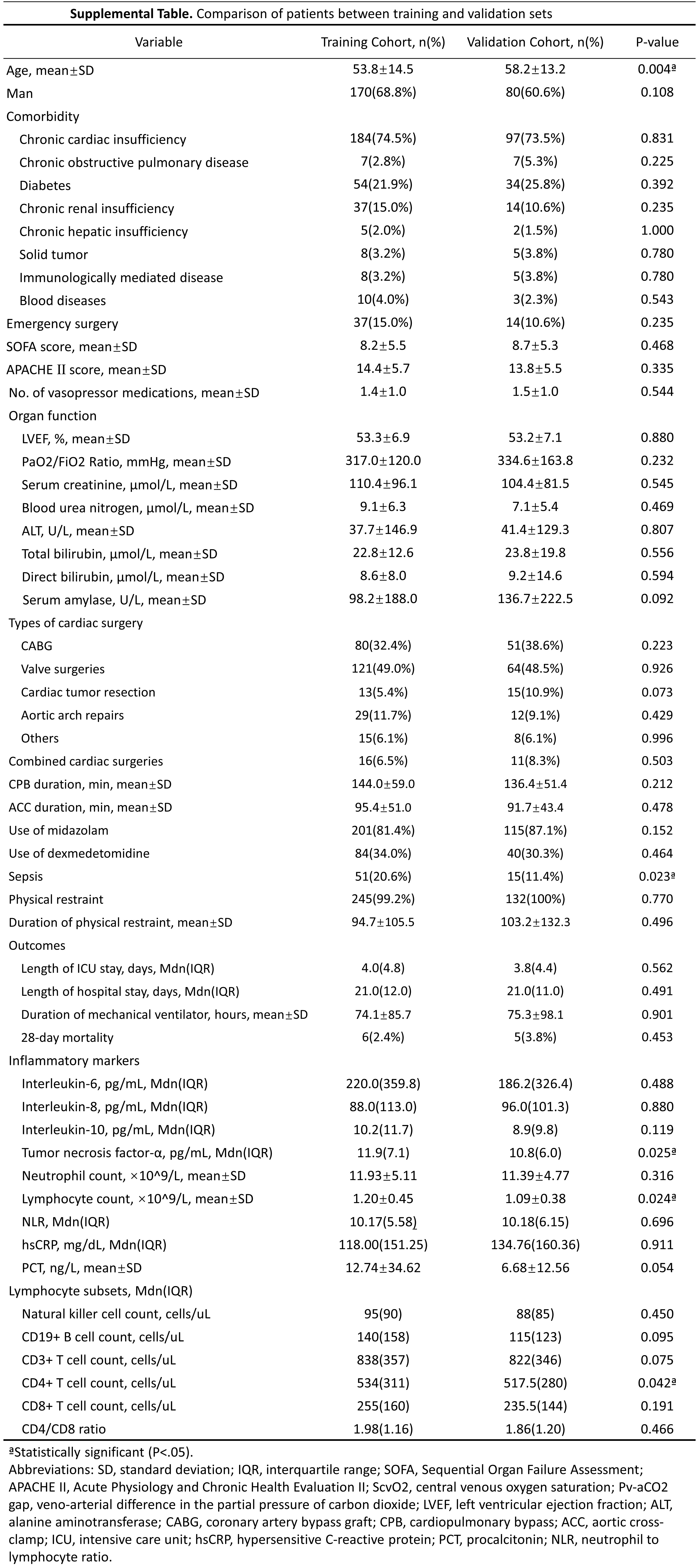

Supplement: Supplementary Table 1 — Comparison of patients between the training and validation sets. [file Image_2.TIF]
